# Biomonitoring of Enniatin B1 and Its Phase I Metabolites in Human Urine: First Large-Scale Study

**DOI:** 10.3390/toxins12060415

**Published:** 2020-06-22

**Authors:** Yelko Rodríguez-Carrasco, Alfonso Narváez, Luana Izzo, Anna Gaspari, Giulia Graziani, Alberto Ritieni

**Affiliations:** 1 Department of Food Chemistry and Toxicology, University of Valencia, Av/Vicent A. Estellés s/n, 46100 Valencia, Spain; 2 Department of Pharmacy, Università di Napoli Federico II, Via D. Montesano, 49-80131 Napoli, Italy; alfonsonsimon@gmail.com (A.N.); luana.izzo@unina.it (L.I.); annagaspari@virgilio.it (A.G.); gulia.graziani@unina.it (G.G.); alberto.ritieni@unina.it (A.R.)

**Keywords:** Enniatin B1, biomonitoring, in vivo, metabolomics, high resolution mass spectrometry (HRMS)

## Abstract

Enniatins (Enns) are mycotoxins produced by *Fusarium* spp. which are a fungus widely spread throughout cereals and cereal-based products. Among all the identified enniatins, Enn B1 stands as one of the most prevalent analogues in cereals in Europe. Hence, the aim of this study was to evaluate for the first time the presence of Enn B1 and its phase I metabolites in 300 human urine samples using an ultrahigh-performance liquid chromatography high resolution mass spectrometry (UHPLC-Q-Orbitrap HRMS) methodology. Enn B1 was detected in 94.3% of samples ranging from 0.007 to 0.429 ng/mL (mean value: 0.065 ng/mL). In accordance with previous in vitro and in vivo analysis, hydroxylated metabolites (78.0% samples) and carbonylated metabolites (66.0% samples) were tentatively identified as the major products. Results from this biomonitoring study point to a frequent intake of Enn B1 in the studied population, suggesting that in-depth toxicological studies are needed in order to understand the potential effects in humans.

## 1. Introduction

Mycotoxins are toxic secondary metabolites mainly produced by the genera *Fusarium*, *Aspergillus*, *Penicillium*, *Claviceps,* and *Alternaria*. These compounds can be found in food and feed commodities, and consuming contaminated products can lead to adverse health effects in humans and animals. *Fusarium* fungi are frequent pathogens of cereal grains and maize, with a large impact in temperate regions of America and Europe [1]. Considering occurrence, toxicity, and consumption data, maximum limits have been set in foodstuffs for several mycotoxins alongside tolerable daily intakes (TDI) or provisional TDIs by a Joint Food and Agriculture Organization and World Health Organization (FAO/WHO) Expert Committee on Food Additives [2]. During the last few years attention has been put into emerging *Fusarium* mycotoxins, such as enniatins (Enns). Its core structure consists of a cyclohexadepsipeptide with alternating residues of three *N*-methyl amino acids and three hydroxyisovaleric acid [3]. To date, 29 different enniatins have been isolated as single compounds or as mixtures of homologues, being Enn B, Enn B1, Enn A1, and Enn A, being the most relevant in that order [4]. These Enns have been reported in cereal samples from Mediterranean and Scandinavian countries in concentration levels ranging from µg/kg to mg/kg [5,6] and also Enns carry-over potential from feedstuffs to animals has been suggested [7,8]. However, no maximum limits have been set for Enns in foodstuffs yet. In 2014, the European Food Safety Authority (EFSA) released a scientific opinion on the risk related to the occurrence of Enns in food and feed, concluding that there might be a concern about chronic exposure but the lack of toxicological studies hampers the risk assessment of dietary exposure [9]. To date, data is still being collected [10].

The toxicity of Enns is based on its ionophoric properties, being able to integrate themselves in biological membranes forming cation selective pores. Transport of mono and divalent cations through these pores disrupts normal physiological concentrations, leading to a wide range of toxicological effects. In vitro studies have reported phytotoxic, insecticidal, antibacterial, enzyme inhibition, antifungal, and immuno-modulatory activities. Besides, cytotoxic effects have been observed in several animal and cell lines at a low micromolar range [4,11]. Nevertheless, Enns have shown low toxicity in vivo, and rapid metabolization and elimination of Enns might be the main reason [12].

Regarding bioavailability, the Enn analogue with the highest oral absorption is Enn B [13,14]. There might also exist a specie-dependent relation, since absolute Enn B1 bioavailability in pigs (91%) is in strong contrast with the one observed in broiler chicken (5%) after single oral application [12,14]. Referring to metabolism, transformation of Enn B1 predominantly occurs via cytochrome P-450 3A-dependent oxidation reaction. Therefore, only phase I products seem relevant, since no phase II metabolites have been found yet. After incubation of Enn B1 with human and pig liver microsomes, 11 different metabolites were structurally characterized using liquid chromatography coupled to: iontrap mass spectrometry (ITMS), multiple-stage mass fragmentation (MS^n^), and high resolution mass spectrometry (HRMS) coupled to an Orbitrap mass spectrometer [15,16]. Biotransformation processes of Enn B1 consisted in carbonylation, carboxylation, hydroxylation, and *N*-demethylation, following the same pattern observed for Enn B except for carbonylation [15,17].

There is scarce literature referring to the detection of enniatins and its resultant metabolites in biological samples, making it harder to assess the risk related to enniatins. Recently, the occurrence of Enn B and its tentative phase I metabolites in human urine was evaluated, finding the parental toxin and some of its tentative metabolites at a concentration of a few ng/mL [18]. Nevertheless, to date, there is no data available concerning the occurrence of Enn B1 and its tentative phase I metabolites in human biological fluids. In order to obtain a risk evaluation precisely, mycotoxins potentially present in foods and its metabolites have to be included in biological studies since the overall toxic profile could be influenced. Therefore, the aim of the present study was to evaluate for the first time the presence of Enn B1 and Enn B1 phase I metabolites in 300 urine samples from volunteers residing in southern Italy using ultrahigh-performance liquid chromatography coupled to high resolution Orbitrap mass spectrometry (UHPLC-Q-Orbitrap HRMS) and to tentatively identify the main Enn B1 metabolic pathway for producing scientific evidence on the pharmacokinetics process of this toxin.

## 2. Results and Discussion

### 2.1. Evaluation of UHPLC-Q-Orbitrap HRMS Conditions

The Enn B1-dependent tandem mass spectrometry (MS/MS) parameters were optimized by injection of 1 µg/mL of Enn B1-analytical standard into the UHPLC-Q-Orbitrap instrument. Results revealed a peak at 8.06 min in full scan mass spectrometry (MS), which showed a stable and abundant ammonium adduct (M + NH_4_)^+^ at *m/z* 671.45986 under positive electrospray ionization (ESI) mode corresponding to Enn B1. The mass error of the observed *m*/*z* was less than 2.5 ppm indicating exceptional agreement with the calculated *m/z.* In addition, product ions (*m/z* 558, 549, 458, 214 and 196) were generated by collision-induced dissociation of the Enn B1 ammonium adduct. These ions correspond to the loss of one or various *N*-methyl-valine, *N*-methyl-isoleucine, and hydroxyisovaleryl residues leading to the formation of main Enn B1 fragments.

Apart from the parent compound, in literature, up to 11 metabolic products were detected and their structures tentatively characterized through high-performance liquid chromatographic/mass spectrometric analyses. In this work, the ammonium adducts of the reported molecular masses were targeted in the Q-Orbitrap HRMS to evaluate the occurrence of the enniatin B1 metabolites in human urine samples. Therefore, a qualitative procedure was developed for detecting theoretical masses of the metabolites previously reported, whereas the same chromatographic gradient was used since all the investigated compounds were able to elute in that short run time.

Data for retention times, observed ion mass, and mass accuracy for Enn B1 and its metabolites are shown in Table 1.

### 2.2. Method Performance

Method results referring to matrix effects, linearity, trueness, repeatability, within-lab reproducibility, limit of quantification (LOQ), and limit of detection (LOD) were obtained following the guidelines set at Commission Decision 2002/ 657/EC [19] are shown in Table 2. A matrix influence was observed, leading to a signal suppression effect (63%) for Enn B1 in the urine samples. Hence, matrix-matched calibration was chosen for quantitative purposes. A coefficient of linearity (R^2^) of 0.9997 was obtained within the range from 0.001 to 5 ng/mL. In order to evaluate the carry-over, a blank sample (*n =* 10) was analyzed just after the highest calibration sample. Since no peaks eluted in the same Enn B1 retention time area, no carry-over was assumed. Acceptable recoveries, ranging from 78% at 0.5 ng/mL to 95% at 5 ng/mL of spiking levels, were obtained. The RSD_r_ and RSD_R_ were ≤12% for all the concentrations studied, remarking the satisfactory precision of the developed method. LOQ and LOD values were extracted from matrix-matched solutions, being 0.0005 and 0.001 ng/mL, respectively. The developed method allowed a reliable detection and quantification of Enn B1 at low ppt range (Table 3).

### 2.3. Occurrence of Enniatin B1 in Human Urines

Enn B1 has been detected in 283 out of 300 urine (94.3%) ranging from <LOQ to 0.429 ng/mL (mean value: 0.065 ng/mL; Table 4). In a previous study, Escrivá et al. [21] evaluated the presence of Enn B1 in urine samples from the Spanish population, reporting occurrence in six samples (60%, *n =* 10), but only two samples could be quantified (0.1–0.34 ng/mL) due to analytical limitations (LOQ = 0.1 ng/mL). Serrano et al. [20] detected Enn B1 in one urine sample (10%, *n =* 10) collected from Italian volunteers, despite having high instrumental sensitivity (LOQ = 0.005 ng/mL). Differences showed among the mentioned studies could be due to the limited sampling. A more recent work, conducted by Liu et al. [22], analyzed 60 urine sample from the Chinese population, reporting the absence of Enn B1 (LOQ = 0.0002 ng/mL). The variation between the Chinese population urinary patterns of Enn B1 and the obtained results could be explained either by dietary habits or the quality of foodstuffs. Wheat has been reported as one of the most susceptible cereals for *Fusarium* spp. contamination [1] and, according to the data reported by FAO [23], the per capita annual consumption of wheat and wheat-based products during 2017 in Italy (146.22 kg) was more than double the consumption in China (62.75 kg). Following the same line, the urinary excretion pattern of Enn B, a structurally similar mycotoxin, was also reported to vary depending on geographical areas with different dietary habits [18]. Recently, strong associations to some cereals and to dietary fiber was reported for Enn B urinary concentration in Sweden [24]. These authors also reported associations to other *Fusarium* mycotoxins, such as deoxynivalenol, in these food categories; nonetheless, Enn B association to rice was negative but strong, which indicates that rice was not as contaminated as the other cereals (e.g., wheat). Hence, if rice consumption was increased in the diet, as in the case of the Asiatic people, the occurrence of Enns in biological fluids would consequently be reduced, which is in line with previous studies [22]. Therefore, the occurrence of Enn B1 in the majority of Italian cereals and cereal-based products analyzed by previous studies [25,26,27] alongside the high consumption of cereals (45–60% kcal per day for an Italian adult) [23] may account for the high incidence reported in the obtained results.

A statistical study for the evaluation of the occurrence of Enn B1 in urines from the three different population groups was conducted. The highest Enn B1 concentration was observed within the low-age group (age ≤ 30 years, mean = 0.071 ng/mL); however, no significant differences were found between occurrence of Enn B1 and age groups (*p* = 0.93). Similarly, gender did not show any correlation referring to Enn B1 concentrations (*p* = 0.85) in accordance with previous mycotoxins monitoring studies in urine [28,29,30].

### 2.4. Urinary Excretion Pattern of Enn B1 Phase I Metabolites

**Figure 1:**
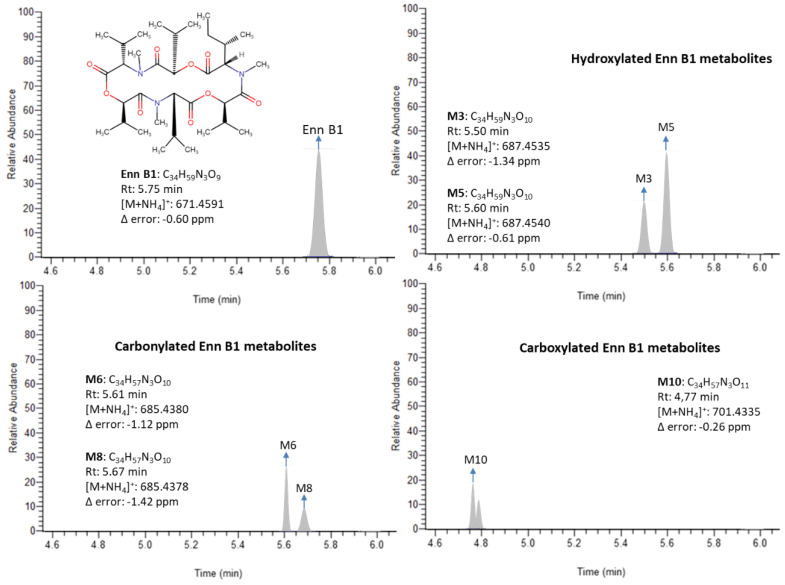
Chromatograms of a human urine sample containing Enn B1 at 0.036 ng/mL; hydroxylated Enn B1 metabolites (M3: 0.053 ng/mL; M5: 0.174 ng/mL); carbonylated Enn B1 metabolites (M6: 0.034 ng/mL; M8: 0.045 ng/mL); and carboxylated Enn B1 metabolites (M10: 0.028 ng/mL; M11: 0.017 ng/mL).

To date, there is scarce literature regarding Enn B1 metabolism. In vivo studies have been carried out in pigs and broiler chickens fed with Enn B1 contaminated feed, reporting the presence up to 11 tentative major Enn B1 metabolites in plasma and showing a good correlation with previous in vitro studies [14,15]. In a recent investigation conducted by Ivanova et al. [16], the same tentative metabolites were detected in vitro after the incubation of human liver microsomes with Enn B1. These metabolites are products of oxidative demethylation (M1), hydroxylation (M2–M5), carbonylation (M6–M8), and carboxylation (M9–M11) reactions. From a chromatographic point of view, all the identified metabolites eluted before the parental compound in reversed-phase chromatography since they became more hydrophilic after going through phase I metabolism pathways (Table 1). Nevertheless, the lack of standards of Enn B1 metabolites avoid an accurate quantification. To overcome that, the matrix-matched calibration curve from the parent compound was used as an approach in order to quantify its metabolites. The results obtained are shown in Table 4. The most prevalent groups were the hydroxylated and carbonylated Enn B1 metabolites, ranging from 0.006 to 0.233 ng/mL (78% samples) and from 0.012 to 1.763 ng/mL (66% samples), respectively. The carboxylated metabolites were found between 0.008 and 0.656 ng/mL (26.3% samples) and the demethylated products ranged from 0.007 to 0.177 ng/mL (5.3% samples). Within the hydroxylated group, the main metabolites were M5 > M3 > M4 > M2, in this order, with M5 representing 68% of all the hydroxylated metabolites quantified. Significant differences were found among concentration values of each metabolite (*p* ≤ 0.05). Similarly, the main carbonylated metabolites were M8 > M6 > M7, and statistical analysis revealed significant differences among them (*p* ≤ 0.05), with M8 being 48% of the total carbonylated metabolites. Whereas results reported by Ivanova et al. [16] showed a similar trend for hydroxylated compounds with M5 as the major one, the most relevant carbonylated product was M6, differing from the here-analyzed samples in which M6 only represented a 32% of the carbonylated products. The most important Enn B1 carboxylated metabolites were M10 and M11, whereas M9 showed very low incidence (0.7%). Ivanova et al. [15] detected M9 and M11 only in in vitro samples of pig liver microsomes, whereas the occurrence of M10 was restricted to in vivo samples. However, the results highlight M11 as another important product of carboxylation pathways in human. Differences observed between this work and in vitro Enn B1 biotransformation assays stand as additional evidence of potential pre-systemic metabolism. The demethylated metabolite M1 appeared to be irrelevant and the multiple reactions needed to generate this product may account for the low incidence showed (5.3%). Figure 1 shows the chromatograms of a sample contaminated with Enn B1 at 0.036 ng/mL; hydroxylated Enn B1 metabolites (M3: 0.053 ng/mL; M5: 0.174 ng/mL); carbonylated Enn B1 metabolites (M6: 0.034 ng/mL; M8: 0.045 ng/mL); and carboxylated Enn B1 metabolites (M10: 0.028 ng/mL; M11: 0.017 ng/mL).

Referring to the samples with no Enn B1 contamination (5.7%, *n =* 17), only 1.3% (*n =* 4) did not show any Enn B1 metabolite, whereas at least two different metabolites were found in the 4.3% (*n =* 13) of the remaining samples, pointing to a complete biotransformation. The results reported above showed that the metabolization of Enn B1 in humans mainly occurs via hydroxylation and carbonylation reactions. Although cytochrome P450 3A4 (CYP3A4) is the main enzyme in the metabolism of Enn B and Enn B1, additional enzymes are also involved; CYP1A2 and CYP2C19 are present in Enn B pathway, whereas CYP3A4/5 is responsible for carbonylated products of Enn B1 as evidenced by Fæste et al. [31] and Ivanova et al. [16]. In fact, a previous work conducted by Rodríguez-Carrasco et al. [18] remarked the hydroxylated and demethylated products as the most relevant Enn B metabolites, whereas demethylated Enn B1 metabolite (M1) showed very low incidence in the present study. Despite a similar pattern of metabolic pathways for Enn B1 and Enn B has been reported in in vitro assays, major metabolites found in human urine are different for each mycotoxin. In the here analyzed samples, major metabolites frequently co-occurred with minor metabolites from the same group; M5 was found alongside any other hydroxylated metabolite in 99% (*n =* 232) of the positive samples (*n =* 234). Referring to carbonylated products, the presence of M8 combined with at least one different metabolite was detected in 52% (*n =* 103) of the positive samples (*n =* 198). Finally, co-occurrence was detected in 34% (*n =* 28) of samples containing carboxylated metabolites (*n =* 79).

## 3. Conclusions

In this study, the occurrence of the *Fusarium* mycotoxin Enn B1 and its phase I metabolites was evaluated in 300 human urine samples throughout ultrahigh-performance liquid chromatography high resolution mass spectrometry (UHPLC-Q-Orbitrap HRMS). Results confirmed the presence of Enn B1 in 94.3% of samples ranging from 0.007 to 0.429 ng/mL (mean value: 0.065 ng/mL). Furthermore, the occurrence of Enn B1 metabolites previously found in in vitro and in vivo analysis was evaluated for the first time in human urine samples. In accordance with literature, demethylated and oxidated metabolite (5.3% samples, mean content = 0.035 ng/mL), hydroxylated metabolites (78.0% samples, mean content = 0.069 ng/mL), carbonylated metabolites (66.0% samples, mean content = 0.196 ng/mL), and carboxylated metabolites (26.3%, mean content = 0.066 ng/mL) were tentatively identified. Statistical analysis confirmed hydroxylated and carbonylated products as the most prevalent metabolites of Enn B1 in human urine. Differences observed between this work and in vitro Enn B1 biotransformation assays stand as additional evidence of potential pre-systemic metabolism. The characterization of metabolites derived from food contaminants is an important issue when performing safety and risk evaluations, and according to the here-obtained results a frequent exposure to Enn B1 is highlighted in Italian population.

## 4. Materials

### 4.1. Chemicals, Reagents and Materials

Methanol (MeOH), acetonitrile (AcN), and water for liquid chromatography (LC) mobile phase (HPLC grade) were purchased from Merck (Darmstadt, Germany). Ammonium formate and formic acid were acquired from Fluka (Milan, Italy). Sodium chloride and C18 were provided from Sigma–Aldrich (Milan, Italy). Syringe filters with polytetrafluoroethylene membrane (PTFE; 15 mm, diameter 0.2 µm) were purchased from Phenomenex (Castel Maggiore, Italy). Analytical standard of Enn B1 (>95% HPLC purity) was obtained from Sigma–Aldrich (Milan, Italy). A stock solution (1 mg/mL in MeOH) was prepared and working standard solutions were built by serial dilution of the stock and stored at −20 °C.

### 4.2. Sampling

During January and February 2018, 300 Italian volunteers residing in Campania region (southern Italy) provided first-spot morning urine samples. The following exclusion criteria were considered for the study: (i) only one member per family allowed; (ii) people with severe issues in liver, kidney, or bile were not allowed due to potential interferences in the metabolic processes related to mycotoxins; (iii) people exposed to high amounts of mycotoxins in a different way from food, such as farmers and veterinarians, were not allowed. The use of medication was not an exclusion criterion due to the lack of information available about interferences with mycotoxins. Urine was stored in plastic containers at −20 °C within 2 h after collection. All volunteers signed informed consent following the Helsinki Declaration on ethical principles for medical research when humans are involved. The present study was accepted by the University of Valencia Institutional human research Committee and the procedures and purposes were properly justified and approved. The numerosity of the sampling (*n =* 300) is in accordance with the International Federation of Clinical Chemists (IFCC) recommendations [32].

Volunteers were asked to specify their age and gender on their own container, in order to classify the sample. The sampling tried to keep the gender parity (male: 45.7%, female: 54.3%). According to the age of participants, three different groups were considered for statistical analysis: ≤30 years old (*n =* 94), from 31 to 59 years old (*n =* 72), and ≥60 years old (*n =* 134). Samples with undetected levels of the analytes of interest were chosen as “blank” and used in spiking and recovery studies. The consumption data were set according to age and gender, following the Guidelines for Healthy Italian Food Supply reported by the National Institute for Food Research and Nutrition [33].

### 4.3. Sample Preparation

The sample was processed following a previous procedure slightly modified [34]. Briefly, 1.5 mL of sample was placed into a 2 mL Eppendorf Safe-Lock Microcentrifuge tube and centrifuged for 3 min at 4000 rpm. After that, 1 mL of the supernatant was transferred to a 15 mL screw cap test tube with conical bottom and 1 mL of acetonitrile was added. The mixture was vortexed for 30 s and subsequently 30 mg of C18 sorbent and 0.3 g sodium chloride were incorporated to minimize interference from matrix. The solution was vortexed again for 30 s and centrifuged for 3 min at 4000 rpm and 4 °C. Then, the upper layer was collected, evaporated under gentle nitrogen stream in a water bath at 45 °C, reconstituted with 0.5 mL of MeOH/H_2_O (70:30 *v*/*v*) and filtered through a 0.2 µm filter before to UHPLC-Q-Orbitrap HRMS analysis.

### 4.4. UHPLC-Q-Orbitrap HRMS Analysis

Quantitative and qualitative profiles of Enn B1 and Enn B1 phase I metabolites were acquired through Ultra High Pressure Liquid Chromatograph (UHPLC; Thermo Fisher Scientific, Waltham, MA, USA) equipped with an auto sampler device, a degassing system, a thermostated (T = 30 °C) Luna Omega 1.6 µm (50 × 2.1 µm) column, a Dionex Ultimate 3000 a Quaternary UHPLC pump working at 1250 bar.

The eluent consisted of two different phases, both H_2_O (phase A) and MeOH (phase B) containing 0.1% formic acid and 5 mM ammonium formate. The gradient elution program for LC prior to Orbitrap HRMS analysis was developed as follows: 0–1 min–0% of phase B, 2 min–95% of phase B, 2.5 min–95% of phase B, 5 min–75% of phase B, 6 min–60% of phase B, 6.5 min–0% of phase B, and 1.5 min–0% phase B for equilibrating the column. The flow rate was set at 0.4 mL/min. A total of 5 µL of the sample was injected. Detection was performed using a Q-Exactive mass spectrometer. Data were acquired through full scan in positive mode at a resolving power of 70,000 FWHM at *m/z* 200. Ion source parameters in positive (ESI+) mode were: sheath gas (N_2_ > 95%) 35, auxiliary gas (N_2_ > 95%) 10, spray voltage 4 kV, capillary temperature 290 °C, auxiliary gas heater temperature 305 °C, S-lens RF level 50. Data analysis and processing were carried-out using the Xcalibur software, v. 3.1.66.10 (Thermo Fisher Scientific, Waltham, MA, USA). A scan range of *m*/*z* 100–800 was set for the compounds of interest; the injection time was set to 200 ms and the automatic gain control (AGC) was selected at 1 × 10^6^. Scan-rate was set at 2 scans/s.

### 4.5. Metabolomic Data Processing

Data processing and data pretreatment were performed to allow the putative identification of significant metabolites. Screening was done by investigating spectral data collected using a mycotoxin spectral library (version 1.1 for Library View Software, AB Sciex, Framingham, MA, USA) containing spectral data for 245 mycotoxins and other fungal/bacterial metabolites and 236 full MS/MS spectral library entries. The features, defined by their m/z and retention time, and their intensities in different samples were used to carry out the statistical analysis. Then, samples were grouped to perform the statistical analysis.

### 4.6. Method Validation

An in-house validation study was conducted according to the Commission Decision 2002/657/EC [19]. The parameters measured included linearity, matrix effect, trueness, precision, LOQ, and LOD. Linearity was evaluated using solvent and matrix-matched calibration curves, analyzing in triplicate six concentration levels ranging between 0.001–5 ng/mL. The matrix-matched calibration curves were prepared spiking aliquots of the corresponding matrices with Enn B1 at similar concentrations than the calibration curve made in solvent. Signal suppression or enhancement effect due to matrix co-elution interferences, was evaluated through a comparison between the slope of pure standard curve with the slope of matrix-matched standard curve following the next equation: SSE (%) = Slope matrix-matched calibration/Slope standard in solvent × 100. Trueness and precision were assessed using recovery studies since no suitable reference material was available. Recovery measurements were performed by spiking blank urines with the standard working solution of Enn B1 at the levels of 0.1, 0.5, 1, and 5 ng/mL. Intra-day (RSD_r_, %) and inter-day precision (RSD_R_, %) were expressed as the relative standard deviation after repeating three measurements per concentration level on the same day and in three non-consecutive days, respectively. LOD was established as the minimum concentration at which the molecular ion can be identified (mass error < 5 ppm) and the LOQ as the lowest concentration of the analyte at which the concentration can be determined with accuracy and precision ≤20%.

### 4.7. Statistical Analysis

Statistical analysis of data was carried out using IBM SPSS version 25 statistical software package (SPSS, Chicago, IL, USA). For comparison of categorical data, the Pearson chi-square and Fisher exact tests were evaluated in order to assess whether Enn B1 occurrence in several subgroups (age, gender, cereal consumption) were significantly different. A non-parametric Kruskal–Wallis test was used to evaluate significant differences in Enn B1 metabolites concentrations. A confidence level of 95 % was assumed for examining data, whereas a *p*-value below 0.05 was considered as significant.

## Figures and Tables

**Table 1:** Retention times, observed mass, and mass accuracy of Enn B1 and its phase I metabolites.

Compound	Retention Time (min)	Molecular Formula	Observed Mass (M + NH_4_)^+^	Accuracy (Δppm)
Enn B1	5.75	C_34_H_59_N_3_O_9_	671.4591	−0.60
M1	4.76	C_33_H_55_N_3_O_11_	687.4175	−0.78
M2	5.44	C_34_H_59_N_3_O_10_	687.4532	−1.78
M3	5.5		687.4535	−1.34
M4	5.55		687.4536	−1.19
M5	5.6		687.4540	−0.61
M6	5.61	C_34_H_57_N_3_O_10_	685.4380	−1.12
M7	5.64		685.4384	−0.54
M8	5.67		685.4378	−1.42
M9	4.1	C_34_H_57_N_3_O_11_	701.4328	−1.26
M10	4.77		701.4335	−0.26
M11	4.79		701.4333	−0.55

**Table 2:** Method performance.

Parameters	R^2^	SSE (%)	Recovery, % (RSD_R_, %; *n* = 9)				LOD (ng/mL)	LOQ (ng/mL)
			5 ng/mL	1 ng/mL	0.5 ng/mL	0.1 ng/mL		
Enn B1	0.9997	63	95 (7)	88 (6)	78 (7)	84 (12)	0.0005	0.001

R^2^, coefficient of correlation; RSD_R_, inter-day relative standard deviation; LOD, limit of detection; LOQ, limit of quantification.

**Table 3:** Available methods for measurement of Enn B1 in human urine.

					Determination		
Urine Samples (*n*)	Origin	Positives Pamples (*n*, (%))	Sample Preparation	Range (ng/mL)	Sensitivity (LOQ, ng/mL)	Detection Method	Reference
10	Italy	1 (10)	SPE	<LOQ	0.005	QQQ (Thermo Fisher Scientific) ESI+ SRM mode	Serrano et al. (2015) [20]
10	Spain	6 (60)	DLLME	<LOQ–0.34	0.1	QQQ (Applied Biosystems) ESI+ SRM mode	Escrivá et al. (2017) [21]
60	China	0 (0)	SPE	-	0.0002	QQQ (AB SCIEX) ESI+ MRM mode	Liu et al. (2020) [22]
300	Italy	283 (94)	SALLE	<LOQ–0.429	0.001	Q-Orbitrap (Exactive, Thermo Fisher Scientific) ESI+ HRMS	This work

ESI+, positive ion mode; HRMS, high-resolution MS; SRM, selected reaction monitoring transition; MRM, multiple reaction monitoring transition; LOQ, limit of quantification; QQQ, triple quadrupole; SPE, solid phase extraction; DLLME, dispersive liquid-liquid microextraction; SALLE, salting-out liquid-liquid extraction.

**Table 4:** Occurrence of Enn B1 and Enn B1 metabolites in the analyzed human urine samples (*n =* 300).

Compound/Group	Incidence (%)	Range (ng/mL)	Mean ^a^ (ng/mL)
Parent Compound			
Enn B1	94.3	0.007–0.429	0.069
Enn B1 Biotransformation Products			
M1	5.3	0.007–0.177	0.035
Demethylated and hydroxylated (M1)	5.3	0.007–0.177	0.035
M2	11.0	0.006–0.019	0.010
M3	50.0	0.005–0.076	0.023
M4	*18.0*	0.002–0.143	0.025
M5	77.3	0.006–0.186	0.047
Hydroxylated group (M2–M5)	78.0	0.006–0.233	0.069
M6	40.0	0.012–1.511	0.105
M7	30.7	0.008–0.510	0.085
M8	48.0	0.042–1.310	0.128
Carbonylated group (M6–M8)	66.0	0.012–1.763	0.196
M9	*0.7*	0.019–0.045	0.032
M10	21.0	0.008–0.241	0.047
M11	14.0	0.002–0.451	0.053
Carboxylated group (M9–M11)	26.3	0.008–0.656	0.066

^a^ Mean values are based in positive samples only.
